# Caseous Intracardiac Calcification: A Diagnostic Enigma

**DOI:** 10.1155/2019/6707690

**Published:** 2019-04-14

**Authors:** Simon G. Findlay, Nicholas M. Child, Douglas F. Muir

**Affiliations:** ^1^North Tees and Hartlepool NHS Trust, Stockton-on-Tees, UK; ^2^The James Cook University Hospital, Middlesbrough, UK

## Abstract

Intramyocardial calcification is a rare phenomenon often only discovered on postmortem. We describe the case of a healthy 69-year-old lady diagnosed with idiopathic caseous intracardiac calcification extending from the mitral valve annulus. We present high-quality images and propose an investigatory template for future cases.

## 1. Introduction

A 69-year-old lady was admitted to the acute medical unit with a several-month history of progressive, exertional breathlessness and atypical chest pain. Cardiac risk factors were hypertension, hyperlipidaemia, and a positive family history of ischaemic heart disease. Drug therapy on admission was simvastatin and amlodipine.

Clinical examination revealed a grade 3 pansystolic murmur radiating to the apex, with no signs of cardiac failure, organomegaly, or lymphadenopathy. The pulse was regular at 81 bpm, BP was 119/90 mmHg, and oxygen saturations were 100% on room air. EKG demonstrated sinus rhythm with fixed, lateral T-wave inversion and ST depression. Serial troponin-Is were 44, 44, and 31 ng/L (normal range: 0-14). Haematology and blood biochemistry were normal including d-dimer, liver function tests, and serum amylase. Chest X-ray demonstrated normal lung fields; however, subtle intracardiac calcification within the cardiac silhouette was visible and comparable to a radiograph in 2015.

A transthoracic echocardiogram (echo) demonstrated mobile valve leaflets with an echo-bright structure in the region of the posterior mitral annulus suggestive of calcification. However, views were limited due to poor echogenicity with incomplete visualisation of the myocardium. A transoesophageal echocardiogram (TOE) revealed a heavily calcified mitral annulus but no mitral stenosis. A bidirectional jet of moderate mitral regurgitation (MR) was noted secondary to extensive mitral annular calcification (MAC) with tethering of the leaflet tips, particularly the anterior mitral valve leaflet. No other valvular heart disease was present, and the left ventricle (LV) retained good contractility throughout with overall normal function.

**Figure 1:**
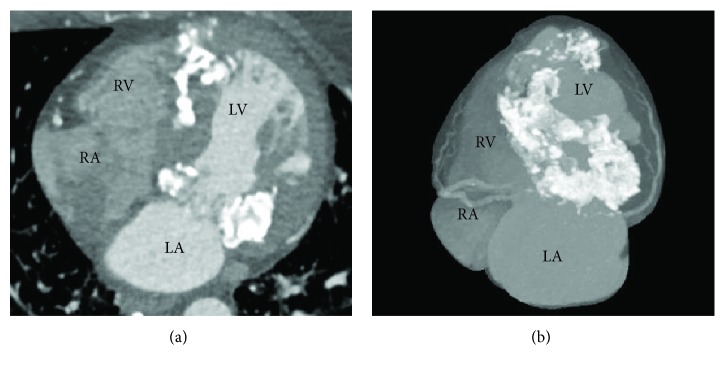
Computed tomography ((a) multiplanar reformatted (MPR) and (b) maximal intensity projection (MIP)) demonstrated extensive infiltrative calcification, circumferentially around the mitral annulus, extending posteriorly and septally into the myocardial apex. RA: right atrium; RV: right ventricle; LA: left atrium; LV: left ventricle.

A CT coronary angiogram (CTCA) (Siemens' SOMATOM Definition Flash CT) showed extensive MAC which infiltrated the left ventricular myocardium resulting in widespread intramyocardial calcification, predominantly in the septum and anterior wall and also in the apex and apical lateral wall (Figure 1). Maximal septal LV wall thickness measured 28 mm and similarly around the infiltrative lesions. The calcified lesions were heterogenous in appearance and extended from the mitral annulus. The external layer of the lesions was calcific, whilst the internal portion was of a heterogenous lower density signal, suggesting caseation. The myocardium contracted normally around the lesions with the estimated ejection fraction of 57%. Calcification of the coronary arteries was apparent with moderate mid-left anterior descending artery (LAD) disease and moderate stenosis of the proximal first diagonal vessel. Thoracic CT screening for extracardiac calcification and carcinoma was unremarkable.

Due to the extent of the intramyocardial calcification, we sought to determine the aetiology and screen for metabolic disease. Serum calcium, phosphate, and eGFR were normal. PTH levels were slightly raised at 8.6 pmol/L (normal range: 1.3-7.3); the vitamin D level was low at 21 nmol/L (normal range: 100-200) indicating vitamin D deficiency. Hb was 119 g/L (normal: 120-160) with negative haemolytic and haematinic screenings. Lipid profile revealed a triglyceride level of 5.0 mmol/L (normal: 0.12-2.10) and HDL of 0.99 mmol/L (normal: 1.2-1.8) with the total cholesterol measuring 5.7 mmol/L. There was an isolated raised bilirubin of 38 microm/L (range: 0-17), but the direct Coombs test and haptoglobin levels did not suggest haemolytic anaemia. Autoantibody screens (immunoglobulins, ANA, ANCA, dsDNA) were negative. Serum iron levels were slightly low; however, the total iron binding capacity was normal. Serum ACE levels were 24 U/L (normal: 8-52). Abdominal ultrasound confirmed fatty liver infiltration. Tuberculosis exposure was excluded through careful history taking and supported with a negative acid-fast bacilli sputum sample.

Although still experiencing exertional breathlessness, the patient's symptoms improved with low-dose furosemide, and given the presence of coronary artery disease on CTCA, she was discharged with dual antiplatelet therapy added to her current medication regimen.

**Figure 2:**
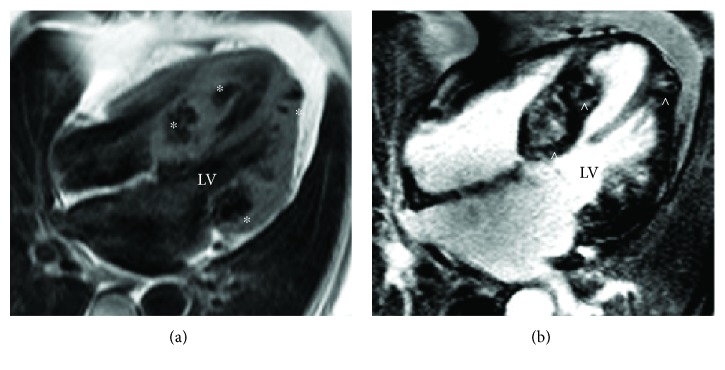
(a) Magnetic resonance T1-weighted spin echo demonstrating multiple low signal lesions throughout the left ventricular myocardium (^∗^), consistent with extensive infiltrative calcification of the myocardium. (b) Magnetic resonance phase-sensitive inversion recovery late gadolinium enhancement demonstrating a heterogenous composition to the intramyocardial lesions within the nulled myocardium (^), suggestive of caseating calcification within the myocardium (four-chamber view Siemens Avanto 1.5 Tesla).

Cardiac MRI was performed (Figure 2), and similar to the CT, this showed extensive calcification extending into the myocardium from the mitral valve annulus, evidenced by the absent signal on T1- and T2-weighted imaging. The lesions enhanced on late gadolinium imaging suggesting the presence of an extracellular matrix within the calcium, but they were not encased entirely by calcium and were in communication with the surrounding myocardium. The findings were consistent with an aggressive caseating, calcific process extending into the left ventricular myocardium from the mitral valve annulus. The infiltrated myocardium was thickened with reduced contractility, whilst the noninfiltrated myocardium contracted well and compensated to maintain an overall normal left ventricular ejection fraction.

**Figure 3:**
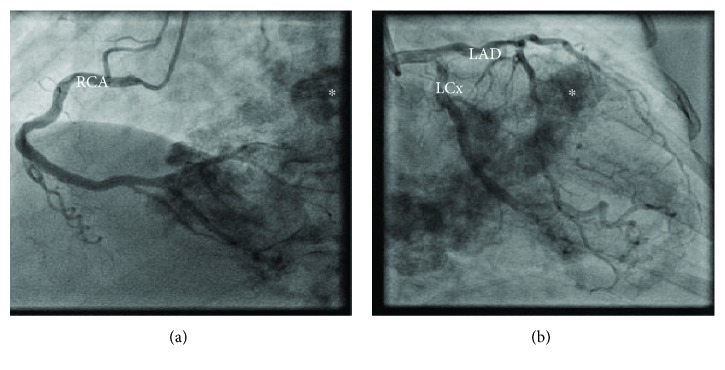
Coronary angiography illustrating the absence of significant obstructive coronary artery disease and highlighting extensive myocardial calcification (^∗^), particularly prominent around the mitral valve annulus: (a) left anterior oblique view and (b) right anterior oblique view. RCA: right coronary artery; LCx: left circumflex artery; LAD: left anterior descending artery.

Coronary angiography showed moderate mid and distal LAD disease, in addition to severe distal posterior descending artery (PDA) disease (Figure 3); this was not felt sufficient to explain the aetiology of her breathlessness.

PET CT was requested to further examine the calcified lesions within the myocardium and evaluate for metastatic calcific phenomenon. This scan demonstrated no abnormal uptake nor metabolic activity outside of the heart, therefore providing reassurance for our patient with exclusion of metastatic infiltrative disease. Interestingly, there was some evidence of metabolic activity around the calcified lesions within the heart; however, it is uncertain whether this represents an active disease process or the chronic inflammatory process described in the caseating tissue.

Given her symptomatic improvement and normal left ventricular function, ongoing surveillance was arranged with interval scanning.

## 2. Discussion

Myocardial calcification may be described in three principle forms: dystrophic, pericarditic, or metastatic. This classification may be useful in identifying the precipitating cause and reaching a unifying diagnosis.

### 2.1. Dystrophic

Dystrophic calcification derives from degenerative myocardial disease, culminating in deposition of calcium salts along the myocardial tissue [1]. Aortic and mitral valves are recognised susceptible areas through their exposure to high degrees of mechanical force and endothelial disruption. This damaged myocardial tissue creates a calcium-philic surface for the calcium salt deposition usually associated with rheumatic heart disease or ageing [2, 3].

Dystrophic calcification can even be divided into endothelial or intramyocardial causes of cardiac endothelial disruption, with ensuing pathological myocardial calcification [3, 4]. Pathologies of myocardial tissue degeneration initiating this endothelial calcification include myocardial infarction, ventricular aneurysms, or preexisting valvular heart disease [3].

Whilst intramyocardial and endothelial calcifications can share identical pathological processes, principally through extensive myocardial ischaemia or mixed valvular heart disease, mechanisms unique to intramyocardial calcification have also been described in the literature. Simonson et al. present a case of myocardial calcification secondary to profound septicaemia, with the acute hypotension triggering diffuse myocardial damage. They hypothesise that causes of catecholamine excess and myofibrillar degeneration can initiate an inflammatory cascade which culminates in intramyocardial calcification [4]. CT can further differentiate these intramyocardial aetiologies with its ability to delineate calcification patterns; infarction produces focal curvilinear calcification within the scarred myocardium, in contrast to systemic sepsis which reveals a typically diffuse picture [4]. CT has even identified rare cases of intramyocardial calcification secondary to hypertrophic cardiomyopathy (HCM), cardiac tumours, and systemic sarcoidosis [1].

### 2.2. Pericarditic

Intramyocardial and pericarditic causes of MAC can be unified through the diagnosis of tuberculosis. Whilst focal myocardial inflammation associated with previous active tuberculosis can become a precursor for calcific change [5], rarer causes of pericarditis-related calcification progressing to the myocardium are attributed to irradiation, ventricular aneurysms, systemic lupus erythematosus (SLE), and viral aetiologies. These patients typically present with pericardial constriction or from incidental imaging findings [6, 7].

### 2.3. Metastatic

Whereas dystrophic and pericarditic calcifications exhibit a normal calcium-phosphate metabolism, metastatic calcification ensues from a derangement of this balance and originates in the previously healthy myocardium [6]. Vascular calcification is a recognised complication of end-stage renal failure, with hyperparathyroidism, hyperuricaemia, and secondary hyperparathyroidism as important contributors. MAC has been demonstrated in patients with renal failure, especially dialysis-dependent patients, and develops due to increased levels of serum calcium caused by hyperphosphataemia. Whilst abnormal calcium-phosphate metabolism due to renal failure accounts for a high incidence of structural cardiac calcification, primary hyperparathyroid hormone excess must first be excluded [8].

Through careful consideration of this classification, we sought to identify the underlying aetiology of our patient's myocardial calcification. Our CT series ruled out pericardial extension and its attributed pathology, however demonstrated caseation of the calcified lesions. Cardiac MRI also suggested an aggressive caseating calcific process extending from the mitral valve annulus. Our images revealed a thickened, infiltrated myocardium with normal surrounding myocardium and overall preserved left ventricular ejection fraction, rather than a restrictive defect seen in endomyocardial fibrosis or pericardial disease [8]. Given these appearances of caseous calcification, a complete medical history and comprehensive serology were obtained therefore excluding inflammatory and metabolic aetiologies of myocardial calcification. Furthermore, imaging with thoracic CT and PET CT helps to rule out infiltrative disease and carcinomatous pathologies.

Cardiac sarcoid is a recognised differential of myocardial calcification; however, the absence of systemic sarcoidosis together with negative serum ACE and normal calcium levels, in addition to our imaging, sufficiently excluded this diagnosis. Increased sensitivity for diagnosing cardiac sarcoid can be achieve through endomyocardial biopsy which reveals a noncaseating granulomatous pattern, whilst cardiac MRI typically demonstrates subepicardial late gadolinium enhancement with extracardiac uptake evidenced on PET CT [9].

The literature identifies dystrophic calcification as the most prevalent aetiology of mitral annular calcification. The presence of calcific deposits extending from our patient's mitral valve annulus is certainly in keeping with dystrophic calcification secondary to degenerative valve disease. However, caseous calcification, a rare variant of mitral annular calcification, has also been recognised with an estimated prevalence of less than 1% of MAC cases [10, 11]. In contrast to the dense calcific deposition of dystrophic calcification, caseous calcification exhibits central areas of lucency within the calcific envelope and is therefore acknowledged as a separate entity. On a cellular level, microscopy has helped further differentiate the two pathologies, with caseous calcification representing an amorphous, acellular mass with a mild chronic inflammatory reaction stimulated by macrophages and lymphocytes. This perhaps explains our CT PET appearances which demonstrated metabolic activity around the calcified lesions. Though echocardiography may suggest the diagnosis of CMAC through identification of echolucency within the calcification, the limited visualisation we experienced ultimately enhanced the value of the advanced imaging modalities utilised and negated the need for biopsy [11, 12].

Cases of intramyocardial calcification are usually discovered on postmortem; therefore, it is rare for us to identify such a case in a minimally symptomatic patient. Strong emphasis should be placed upon establishing the underlying cause of intramyocardial calcification and differentiating the unifying diagnosis from cardiac tumours, myocardial abscesses, or hydatid cysts, given their prognostic implications. In the case of caseous MAC (CMAC), complications of arrhythmia, coronary events, and sudden cardiac death though rare intensify its significance as a diagnostic and prognostic entity [10, 12].

Despite establishing the diagnosis of “idiopathic intramyocardial calcification with caseous mitral annular calcification,” the pertinent question remains: *How can we best treat our patients?* No consensus has been reached on the management of CMAC; one school of thought favours conservative management given the dynamic physiological yet benign process and reported spontaneous resolution of intramyocardial calcification, whilst the other favours surgical decalcification and even mitral valve replacement especially in the context of symptomatic valvular pathology [10, 11, 13]. Dietl et al. even makes the case for elective surgical resection of CCMA in asymptomatic patients [14]. Their review evaluated the prevalence of cerebral embolisation associated with CMAC, with CMAC patients at an increased risk of cardioembolic stroke (19.2%) especially when compared to MAC patients (11.8%, *p* = 0.018). This may originate from spontaneous fistulisation and embolisation of caseous necrotic material from the CMAC lesion, rendering anticoagulation ineffective for prevention of cardioembolic stroke [14]. With the ever increasing availability and value of cardiac CT, we expect many physicians to face the challenge of managing this complex condition; therefore, we aim to stimulate further research into the optimal management. For our patient, given the presence of coronary artery disease, we commenced antiplatelet therapy, continued primary cardiovascular prevention with the aim of attenuating the calcific process, and continued with diuretic therapy. She remained stable on clinical follow-up and is awaiting interval scanning.

Our case illustrates well the diagnostic enigma of idiopathic intramyocardial calcification. The atypical presentation coupled with the inconclusive first-line investigations posed a diagnostic dilemma. Furthermore, despite establishing a diagnosis of CMAC-associated mitral regurgitation, identifying the aetiology and improving patient symptoms provided additional clinical challenge. This manuscript describes our experience of idiopathic intramyocardial caseous calcification which should guide physicians encountering this condition, as well as stimulate further research into this rare, though fascinating, diagnosis.
